# *Notes from the Field:* Adverse Event Associated with Unintentional Exposure to the *Brucella abortus* RB51 Vaccine — Oregon, December 2017

**DOI:** 10.15585/mmwr.mm6726a4

**Published:** 2018-07-06

**Authors:** Sarah M. Hatcher, David Shih, Jacobey Holderman, Caitlin Cossaboom, Richard Leman, Emilio DeBess

**Affiliations:** ^1^Epidemic Intelligence Service, CDC; ^2^Northwest Tribal Epidemiology Center, Northwest Portland Area Indian Health Board, Portland, Oregon; ^3^Public Health Division, Oregon Health Authority, Portland, Oregon; ^4^Baker County Health Department, Oregon; ^5^Division of High Consequence Pathogens and Pathology, National Center for Emerging Zoonotic and Infectious Diseases, CDC.

On December 7, 2017, a previously healthy, middle-aged male veterinarian was evaluated at an Oregon emergency department (ED) for cough, malaise, myalgia, fever, and arthralgia of 4 days’ duration. The patient reported having sustained a needle stick while administering the *Brucella abortus* strain RB51 vaccine (RB51) to cattle 3 weeks before symptom onset. While the patient was in the ED, a probable diagnosis of brucellosis was considered, but *Brucella* testing was not performed. After a chest radiograph, the patient was discharged with a doxycycline prescription for right upper lobe pneumonia. On December 11, the patient returned to the ED with worsening pneumonia. At that time, the Oregon Health Authority Public Health Division (OPHD) was notified of the probable brucellosis case. The patient was hospitalized and began oral rifampin and intravenous ceftriaxone and azithromycin, and continued oral doxycycline treatments. OPHD and the local health jurisdiction provided RB51-specific treatment and testing recommendations to clinicians and provided guidance for laboratory biosafety precautions through coordination with the Oregon State Public Health Laboratory. As a result, the hospitalist discontinued rifampin, continued doxycycline, and started trimethoprim-sulfamethoxazole (TMP/SMX). By 3 days after admission, the patient’s symptoms had improved, and he was discharged and prescribed doxycycline and TMP/SMX for 60 days, which is the recommended treatment for human RB51 infections (*1*). Blood and sputum cultures collected at admission were later negative for *Brucella spp*. During reinterview, the patient confirmed that his only known RB51 exposure was the needle stick. Although he administered the vaccine regularly and was aware of its potential for pathogenicity in humans, he had not sought the recommended postexposure prophylaxis of doxycycline and TMX/SMX for 21 days (*1*).

Brucellosis is a zoonotic bacterial disease of humans and many animal species, with a low infectious dose in humans (*1*). Occupational *Brucella spp.* exposures most commonly affect veterinarians, health care workers, and laboratorians. RB51 is a live-attenuated vaccine, approved for use as part of the Brucellosis Eradication Program in the mid-1990s (*2*). It is resistant to rifampin, a first-line treatment choice for human brucellosis, and does not induce an antibody response detectable by commercially available serologic assays, requiring culture for confirmation (*1*). Human infections with RB51 most commonly result from needle-stick injuries, which are relatively common and underreported among veterinarians (*3*). In a summary of RB51 exposures reported to CDC during 1998–1999, seven of 26 (27%) persons reported persistent illnesses with symptoms similar to those reported by this patient (*2*). Although killed by pasteurization and not commonly shed in milk, RB51 recently gained attention nationwide during investigation of cases and exposures after raw (unpasteurized) milk consumption in Texas and New Jersey (*4*,*5*). These cases highlighted the lack of awareness of the unique challenges in diagnosing and treating RB51 infections in humans.

This report serves as a reminder that occupational RB51 exposure is a risk among veterinary personnel. Clinicians, laboratory staff members, and public health officials should be aware of RB51 diagnosis and treatment challenges and be prepared to manage RB51 cases and exposures. State and local health jurisdictions should consider regular communication with veterinary and laboratory communities regarding occupational RB51 exposures and can serve as a resource to clinicians unfamiliar with management of human RB51 *Brucella* infections and exposures.
